# Cost analysis of UMMC services: estimating the unit cost for outpatient and inpatient services

**DOI:** 10.1186/1472-6963-12-S1-O1

**Published:** 2012-11-21

**Authors:** Maznah Dahlui, Ng Chiu Wan, Tan Seow Koon

**Affiliations:** 1 Julius Centre, department of Social and Preventive Medicine, Faculty of Medicine, University of Malaya, Kuala Lumpur, Malaysia; 2 University of Malaya Medical Centre, Kuala Lumpur, Malaysia

## Introduction

Hospital cost analysis is an essential tool relating the inputs of resources in monetary terms to the outputs of services provided by hospitals. Cost information is part of the basic information needed by hospital managers and national policy makers to enable them to make informed decisions to enhance performance of hospitals in a health care delivery system and for efficient allocation of resources within or between hospitals.

## Objectives

The primary objective of the study was to determine the actual costs of health care service provision, including costs for surgical procedures, at the University of Malaya Medical Centre (UMMC) for the calendar year 2010. The UMMC is the academic teaching hospital of the University of Malaya and also a major tertiary referral hospital in Malaysia.

## Methods

The study applied a top-down method and step-down allocation of overhead costs to the final health care departments. The categories of costs included in the estimation included costs for personnel, overheads and the annualized costs of equipment associated with the treatment of patients on an aggregate as well as a per capita basis. The final study outputs were expressed as cost per visit for outpatient and daycare services as well as the cost per diem and per admission for medical and surgical inpatient services.

## Results

The average length of hospital stay (ALOS) for all admissions in 2010 was 6.30 days (SD 8.945) while the ALOS for the medical and surgical wards were 6.7 days (SD 8.886) and 5.6 days (SD 9.005) respectively. The costs per admission at the medical and surgical wards were RM 4,296 and RM 6,073.71 respectively, while the cost per diem were RM 641.15 and RM 1,085.48, respectively. The average cost for a surgical procedure performed at the operating theatre was RM 1084.59. The major cost component for the medical wards was for consumables which made up 70% of the total cost for the medical inpatient services. In contrast, costs for treatment procedures made up 62% of total costs for the surgical inpatient services. Although there were more medical wards and more medical admissions compared to surgical wards and admissions, there was no significant difference in the proportion of staff emoluments to the total cost for medical and surgical inpatient services (approximately 11%). The costs per outpatient and day-care visit were RM 239.19 and RM 777.24 respectively. The proportion for consumables at the outpatient was half than that of day-care; consistent to the type of healthcare services given at day-care.

## Conclusion

The costs per diem for medical inpatient services were 1.7 times higher than surgical services. The costs per visit for outpatient services were 3.2 times higher than daycare. The study results are of use in future economic evaluation studies in the UMMC to help identify the most cost-effective modality of treatment for specific diseases as well as in the determination of cost weights for case-mix hospital reimbursement system in a major academic teaching hospital.

